# Xiao-Chai-Hu Decoction Ameliorates Poly (I:C)-Induced Viral Pneumonia through Inhibiting Inflammatory Response and Modulating Serum Metabolism

**DOI:** 10.1155/2022/1240242

**Published:** 2022-07-12

**Authors:** Feng Chen, Fei Qu, Yuehui Jia, Chengxin Wang, Yuejie Yu, Jiabao Liao, Min Lin, Fengjuan Chen, Zhijia Sun

**Affiliations:** ^1^Jiaxing Hospital of Traditional Chinese Medicine, Jiaxing, China; ^2^The Third Affiliated Hospital of Guangzhou University of Chinese Medicine, Guangzhou, China

## Abstract

Viral pneumonia is widespread, progresses rapidly, and has a high mortality rate. Developing safe and effective therapies to treat viral pneumonia can minimize risks to public health and alleviate pressures on the associated health systems. Xiao-Chai-Hu (XCH) decoction can be used in the treatment of viral pneumonia. However, the mechanisms of XCH on viral pneumonia remain unclear. In this study, poly (I:C) was used to establish a mouse model of viral pneumonia, and the therapeutic effects of XCH on viral pneumonia were assessed. Furthermore, we evaluated the effects of XCH on inflammatory response. Lastly, untargeted metabolomics were used to study the metabolic regulatory mechanisms of XCH on viral pneumonia model mice. Our results showed that XCH treatment decreased the wet/dry ratio in lung tissue, total protein concentration, and total cell count in bronchoalveolar lavage fluid (BALF). H&E staining indicated that XCH treatment alleviated the pathological changes in lung. Moreover, XCH treatment decreased the levels of proinflammatory cytokines (IL-1*β*, IL-6, and TNF-*α*) and lowered the ratio of CD86^+^/CD206^+^ macrophages and CD11b^+^LY6G^+^ neutrophils in BALF. XCH treatment also decreased the myeloperoxidase (MPO) and reduced the phosphorylations of PI3K, AKT, and NF-*κ*B p65 in lung. Serum untargeted metabolomics analysis showed that XCH treatment could affect 18 metabolites in serum such as creatine, hydroxyproline, cortisone, hydrocortisone, corticosterone, hypotaurine, and taurine. These metabolites were associated with arginine and proline metabolism, steroid hormone biosynthesis, and taurine and hypotaurine metabolism processes. In conclusion, our study demonstrated that treatment with XCH can ameliorate viral pneumonia and reduce inflammatory response in viral pneumonia. The mechanism of action of XCH in the treatment of viral pneumonia may be associated with inhibiting the activation of PI3K/AKT/NF-*κ*B signaling pathway in lung and regulating arginine and proline metabolism, steroid hormone biosynthesis, and taurine and hypotaurine metabolism in serum.

## 1. Introduction

Viral pneumonia is widespread, progresses rapidly, and has a high mortality rate. The outbreak of coronavirus disease 2019 (COVID-19) once again demonstrates that not only are respiratory viruses a serious threat to human life globally but also they have a huge impact on international production and commerce [1]. Acute lung injury and acute respiratory distress syndrome (ARDS) are the main causes of death in viral pneumonia patients [2]. The high variability of viruses and complex pathogenic mechanisms involved severely restricts the research and development of vaccines and drugs. Therefore, developing safe and effective therapies against novel respiratory viruses can minimize risks to public health and alleviate pressures on the associated health systems [3].

Currently, respiratory viruses are believed to cause hyperactivation of immune cells in the lung, resulting in cytokine storm, which is the main pathophysiological basis of viral pneumonia [4]. A cytokine storm is an excessive immune response produced by the body to external stimuli, such as viral infection, and is characterized by high levels of immune cell activation and subsequent production of large amounts of cytokines and chemical mediators [5]. Viral infection can cause lung tissues to secrete chemokines to stimulate immune cells, such as macrophages and neutrophils, to migrate toward the lungs. Simultaneously, viruses can also cause hyperactivation of immune cells in lung tissues and production of high levels of cytokines such as TNF-*α*, IL-1*β*, and IL-6. These proinflammatory cytokines can activate more immune cells and cause severe lung injury, ARDS, or even death [6].

Traditional Chinese medicine (TCM) has been demonstrated with significant effects in the treatment of COVID-19 [7]. In the treatment of COVID-19 using TCM, “three medicines and three formulas” were obtained through clinical screening [8]. These included Jin-Hua-Qing-Gan granules, Lian-Hua-Qing-Wen capsules, Xue-Bi-Jing injection, Qing-Fei-Pai-Du decoction, Hua-Shi-Bai-Du decoction, and Xuan-Fei-Bai-Du decoction, which were included in the “Diagnosis and Treatment Protocol for COVID-19 (Interim 8th Edition)” and can effectively decrease the incidence and progression and mortality rates of COVID-19 and lead to a promotion in negative result of nucleic acid amplification detection, improvement of the cure rate, and acceleration of recovery [9]. Xiao-Chai-Hu (XCH) decoction consists of *Bupleurum chinense* DC., *Scutellaria baicalensis* Georgi, *Panax ginseng* C. A. Mey., *Pinellia ternata* (Thunb.) Makino, *Glycyrrhiza glabra* L., *Zingiber officinale* Roscoe, and *Ziziphus jujuba* Mill. and has anti-infective and antiviral effects [10, 11]. Besides, XCH is a basic formula in Qing-Fei-Pai-Du decoction used for treating COVID-19. A data-mining study based on the records of traditional Chinese medicine showed that XCH can be the potential drug on COVID-19 [12]. However, the underlying mechanisms of XCH on viral pneumonia are unknown. In this study, poly (I:C) was used to establish a mouse model of viral pneumonia, and the therapeutic effects of XCH on viral pneumonia were assessed. Furthermore, we evaluated the effects of XCH on inflammatory response. Lastly, untargeted metabolomics were used to study the metabolic regulatory mechanisms of XCH on viral pneumonia model mice.

## 2. Materials and Methods

### 2.1. Reagents

Polyinosinic-polycytidylic acid (poly (I:C)) (CAS No. 24939-03-5; Batch No. S18188) was purchased from Shanghai Yuanye Bio-Technology Co., Ltd. (Shanghai, China). Dexamethasone (DXM) (CAS No. 50-02-2; Batch No. D8040) was purchased from Solarbio Biotechnology Co., Ltd. (Beijing, China). Total protein assay kit (Batch No. A045-4-2) and myeloperoxidase (MPO) (Batch No. A044-1-1) assay kit were obtained from Nanjing Jiancheng Biological Engineering Institute (Nanjing, China). Enzyme-linked immunosorbent assay (ELISA) kits of mouse tumor necrosis factor alpha (TNF-*α*) (Batch No. EK282), interleukin-1*β* (IL-1*β*) (Batch No. EK201B), and IL-6 (Batch No. EK206) were purchased from Multi Science Biotechnology Co., Ltd. (Hangzhou, China). PE anti-mouse CD86 antibody (Batch No. 105008), APC anti-mouse CD206 antibody (MMR) (Batch No. 141708), APC anti-mouse Ly6G antibody (Batch No. 127614), and PE anti-mouse CD11b antibody (Batch No. 101208) were purchased from BioLegend, Inc. (Beijing, China). Rabbit anti-PI3-kinase kinase (PI3K) antibody (Batch No. bs-10657R) and rabbit anti-phospho-PI3-kinase p110 beta (Ser1070) (p-PI3K) antibody (Batch No. bs-6417R) were purchased from Beijing Bioss Biotechnology Co., Ltd. AKT monoclonal antibody (Batch No. 60203-2-Ig), phospho-AKT (Ser473) monoclonal antibody (Batch No. 66444-1-Ig), nuclear factor *κ*B (NF-*κ*B) p65 polyclonal antibody (Batch No. 10745-1-AP), and *β*-actin polyclonal antibody (Batch No. 20536-1-AP) were purchased from Proteintech Group, Inc. (Wuhan, China). Phospho-NF-*κ*B p65 (Ser536) (Batch No. #3033) monoclonal antibody was purchased from Cell Signaling Technology, Inc. (Shanghai, China). Goat anti-rabbit IgG H&L (HRP) (Batch No. ab205718) and goat anti-mouse IgG H&L (HRP) (Batch No. ab205719) were purchased from Abcam Co., Ltd. (Shanghai, China). Reference standards of saikosaponin A (CAS No. 20736-09-8; Batch No. R015101), saikosaponin D (CAS No. 20874-52-6; Batch No. R015102), and baicalin (CAS No. 21967-41-9; Batch No. R096642) were purchased from Sichuan Weikeqi Biological Technology Co., Ltd. (Sichuan, China).

### 2.2. Experimental Animals

Specific pathogen free- (SPF-) grade adult male C57BL/6 mice weighing 20 ± 2 g were purchased from Beijing HFK Bio-Technology Co., Ltd. (qualification certificate no. SCXK (Beijing) 2020-0004). Mice were housed in a SPF-grade clean environment with five mice per cage at a temperature of 22 ± 2°C, humidity of 50% ± 15%, and a light-dark cycle of 12 h/12 h; mice were given ad libitum access to food and water. All procedures were approved by the Institutional Animal Care and Use Committee of Jiaxing Hospital of Traditional Chinese Medicine.

### 2.3. Establishment of a Mouse Model of Viral Pneumonia

Poly (I:C) is a double-stranded ribonucleic acid and is commonly used as a viral mimic in many studies [13, 14]. The viral pneumonia mouse model was induced using poly (I:C) based on the previous study [15]. Briefly, after isoflurane was used for basal anesthesia, the incisors and limbs of the mouse were fixed facing up at a 60° angle on the mouse board and placed in a clean bench with the head elevated and the tail lowered. The neck was fully exposed and disinfected, and the trachea was exposed layer by layer. A 1 mL syringe was used to aspirate poly (I:C), and the left hand slightly fixed the position of the trachea. The needle was inserted into the trachea diagonally in the concentric direction. When an empty space was felt, the needle was lowered and partially inserted in a slightly horizontal manner. The lack of resistance on suction and presence of air indicate that the needle was in the trachea. Poly (I:C) (5 mg/kg, dissolved in 50 *μ*L PBS) was slowly injected into the trachea before a small volume of air was finally injected to ensure that all of the drug had entered the trachea.

### 2.4. Preparation and Quality Control of XCH

30 g of *Bupleurum chinense* DC. (Batch No. 210103), 9 g of *Scutellaria baicalensis* Georgi (Batch No. 201217), 9 g of *Panax ginseng* C. A. Mey. (Batch No. 200811), 9 g of *Pinellia ternata* (Thunb.) Makino (Batch No. 201030), 9 g of *Glycyrrhiza glabra* L. (Batch No. 210124), 9 g of *Zingiber officinale* Roscoe (Batch No. 200815), and 12 g of *Ziziphus jujuba* Mill. (Batch No. 201002) were weighed and soaked with an eightfold volume of water. Then, the mixture was decocted for 30 min twice and concentrated into 5 g of crude herb/mL. All herbs were purchased from Tianjin Traditional Chinese Medicine Prepared Pieces Co., Ltd. and authenticated by pharmacist of the Jiaxing Hospital of Traditional Chinese Medicine.

High performance liquid chromatography (HPLC) (UltiMate 3000) coupled with mass spectrometer (MS) (Q Exactive™) was used to conduct the quality control of XCH (Thermo Scientific, USA). Briefly, the test solution of XCH was injected onto an Eclipse Plus C18 RRHD column (2.1 × 100 mm, 1.8 *μ*m; column temperature: 40°C; flow rate: 0.3 mL/min; injection volume: 5 *μ*L). Mobile phase A was 0.1% formic acid aqueous solution. Mobile phase B was acetonitrile. The mobile phase condition was as follows: 5% B, 0 min; 5% B, 1 min; 80% B, 9 min; 100% B, 12 min; 100% B, 14 min; 5% B, 14.1 min; and 5% B, 16 min. Mass spectrum equipped with an electrospray ionization (ESI) source was performed for both positive and negative ionization scan modes. The m/z ranges from 100 to 1,500 Da. The spray voltage was 3,500 V (positive mode) and 3,000 V (negative mode), respectively. The capillary temperature was 320°C, the sheath gas-flow rate was 30 arbitrary units, and the auxiliary gas-flow rate was 10 arbitrary units in both positive and negative modes. Saikosaponin A, saikosaponin D, and baicalin were used as the reference standards for the quality control of XCH.  Figure S1 shows the typical based peak intensity (BPI) chromatograms of XCH and reference standards (saikosaponin A, saikosaponin D, and baicalin) and Table S1 exhibits the characteristic fragment ions of reference standards.

### 2.5. Experiment Grouping and Dosing Regimen

Fifty mice were acclimatized for 1 week before they were randomized into five groups: control, model, positive control, and XCH low- and high-dose groups. Among the groups, intratracheal injection of poly (I:C) in the model, positive control, and XCH low- and high-dose groups was used to construct the viral pneumonia model, while 50 *μ*L PBS was administered to the control group by intratracheal injection. After successful model construction, 0.2 mL of physiological saline was administered to the control and model groups once every 12 h. DXM at 0.5 mg/kg was administered once via intraperitoneal injection to the positive control group [16], while 11.31 and 22.62 crude herb/kg were orally administered once every 12 h to the XCH low- and high-dose groups, respectively. The XCH dose was obtained by using the conversion formula for the daily dose in a human to the animal dose, whereas the dose used for the low-dose group was the equivalent human dose. At 24 h after model construction and administration, sodium pentobarbital (50 mg/kg) was administered via intraperitoneal injection to anesthetize the mice, and a syringe was used to collect blood from the abdominal aorta. The collected blood was centrifuged at 3,000 rpm for 15 min to collect serum.

### 2.6. Collection of Bronchoalveolar Lavage Fluid (BALF)

After serum was collected, mice were euthanized, and the thoracic cavity was opened. The cervical trachea was exposed layer by layer and a 1-2 cm incision was made in the trachea. The lavage needle was inserted in the bottom of the right bronchus, and a surgical suture was employed to suture the trachea and lavage needle tightly. A surgical suture was used to ligate the left lung hilum to ensure that the left lung was sealed. A volume of 1 mL physiological saline was aspirated using a syringe and connected to the lavage needle at the cervical trachea. Physiological saline was slowly injected into the right lung to remain in the alveoli for 15–30 s. During this period, physiological saline was closely monitored for exudation. After that, the lung was gently massaged, and BALF removed by suction. This process was repeated three times, and a total of 2.5 mL of BALF was collected from each mouse.

### 2.7. Measurement of Wet/Dry Ratio of Lung Tissue

After BALF was collected from each mouse, a portion of the left lung tissue was collected and weighed. The wet tissue weight (*W*) was measured, and then the tissue was dried in an 80°C thermostatic oven for 48 h until lung weight no longer decreased, which was taken as the dry weight (*D*). The *W*/*D* ratio of the lung tissue was calculated and used to measure the severity of lung edema.

### 2.8. Hematoxylin and Eosin (H&E) Staining of Lung Tissue

A portion of the left lung tissue was collected and mixed with formalin. This was followed by paraffin embedding, sectioning into 3 *μ*m section, routine H&E staining, and neutral resin mounting. Histopathological changes in lung tissues of every group were observed under a microscope.

### 2.9. Measurement of Total Protein Concentration in BALF

The collected BALF was centrifuged (4°C, 3,000 rpm, 10 min), and the supernatant was collected. A total protein assay kit was used to measure total protein concentration in BALF.

### 2.10. Measurement of Total Cell Count in BALF

A total of 200 *μ*L PBS was added to the precipitate of BALF for resuspension; 20 *μ*L of the resuspended cells was used for calculating the total cell count on a cell counting chamber.

### 2.11. Measurement of Proinflammatory Cytokines in BALF Using ELISA

The levels of inflammatory factors (IL-6, IL-1*β*, and TNF-*α*) in collected BALF supernatant were measured using ELISA kits according to the manufacturers' instructions.

### 2.12. Flow Cytometry Analysis of Macrophage Polarization and Neutrophil Expression in BALF

The collected BALF was centrifuged (4°C, 3,000 rpm, 10 min), and the precipitate was retained and then divided equally into two parts. PE anti-mouse CD86 and APC anti-mouse CD206 antibodies (1 *μ*L each) were added to the first cell pellet to label macrophages, and APC anti-mouse Ly6G and PE anti-mouse CD11b antibodies (1 *μ*L each) were added to the second cell pellet to label neutrophils. Negative and blank controls were set up at the same time for all cell staining. The flow cytometry antibodies were gently mixed with cells and then incubated at 4°C and in the dark for 30 min. After incubation, samples were analyzed using a flow cytometer (BD Biosciences, Franklin Lakes, NJ, USA), and macrophage polarization and neutrophil expression were analyzed with FlowJo software.

### 2.13. Measurement of MPO Activity in Lung Tissue Homogenates

50 mg of lung tissues was weighed and mixed with 450 *μ*L of normal saline and homogenized on ice. Then, the homogenized mixture was centrifuged at 3,000 rpm/min for 15 min and the supernatant was collected to obtain the tissue homogenate. The activity of MPO in lung tissue homogenate was detected based on the manufacturers' instructions.

### 2.14. Western Blot

The protein levels of PI3K/AKT/NF-*κ*B signaling pathway were detected using western blot based on the previous study [17]. Briefly, 50 mg of lung tissues was weighed, immersed into RIPA lysis buffer, and lysed for 30 minutes on ice and then centrifuged at 12,000 rpm/min for 10 min, and the supernatant was collected and the protein concentration in supernatant was detected using test kit. 20 *μ*g of protein samples in each group was added to an 8–12% sodium dodecyl sulfate polyacrylamide gel for electrophoresis and transferred to methanol-activated polyvinylidene difluoride (PVDF) membranes. Then, membranes were blocked using 5% skim milk for 2 hours, after which they were incubated with anti-PI3K (1 : 1,000), p-PI3K (1 : 1,000), AKT (1 : 1,000), p-AKT (1 : 1,000), NF-*κ*B p65 (1 : 2,000), p-NF-*κ*B p65 (1 : 2,000), and *β*-actin (1 : 5,000) at 4°C overnight. On day 2, membranes were washed with 1 × TBST buffer for three times for 10 min each and incubated with corresponding secondary antibodies for 2 h. After incubation of secondary antibodies, membranes were washed with 1 × TBST buffer for three times for 5 min each. Blotting was visualized using enhanced chemiluminescence method on a gel imaging system. *β*-Actin was the internal control. ImageJ software was used to quantify the gray values of each band and the relative protein expression was calculated based on the gray values.

### 2.15. Untargeted Metabolomic Analysis

Untargeted metabolomic analysis was conducted based on the previous study [16].

### 2.16. Sample Preparation

A total of 400 *μ*L 80% methanol solution was added to 100 *μ*L serum sample, and the solution vortexed. The solution was then incubated in an ice bath for 5 min before being centrifuged (20 min, 15,000 × *g*, 4°C). After centrifugation, ultrapure water was added to dilute the supernatant until the methanol content was 53%. The solution was then centrifuged at 15,000 × *g* and 4°C for 20 min. The supernatant was collected and used as a test sample. An equal volume of each sample was taken and mixed to obtain the quality control (QC) sample.

### 2.17. Chromatography and Mass Spectrometry Conditions

A Hypersil GOLD (C18) chromatography column (2.1 × 100 mm, 1.9 *μ*m) was used for chromatography. The mobile phases were 0.1% formic acid (A) and methanol (B). The gradient elution used was as follows: 0 min, 98% A, 2% B; 1.5 min, 98% A, 2% B; 12 min, 0% A, 100% B; 14 min, 0% A, 100% B; 14.1 min, 98% A, 2% B; and 17 min, 98% A, 2% B. Column temperature was 40°C; flow rate was 0.2 mL/min; and sample volume was 2 *μ*L.

For mass spectrometry, electrospray ionization (ESI) positive and negative ion modes were simultaneously used for detection. The ESI source settings were as follows: spray voltage of 3.2 kV; sheath gas-flow rate of 40 arb; aux gas-flow rate of 10 arb; capillary temperature of 320°C. Polarity was positive: negative. The scan range was 100–1500 m/z. QC samples were added after every 6 samples to assess experiment stability.

### 2.18. Data Processing and Analysis

High-resolution mass spectrometry was used to detect the molecular characteristic peaks of samples. Matching and identification were performed for the molecular characteristic peaks of samples by using the high-quality mzCloud, mzVault, and MassList databases constructed using standards. The source file obtained from mass spectrometry (raw) was inputted into Compound Discoverer 3.1 (CD3.1, Thermo Fisher) software for data preprocessing. First, the retention time and *m/z* ratio were used for simple screening. Following that, peak alignment was performed for different samples based on a retention time deviation of 0.2 min and mass deviation of 5 parts per million (ppm) to ensure accurate identification. Following that, peak extraction was performed using device settings of 5 ppm, signal intensity deviation of 30%, signal-to-noise ratio of 3, minimum signal intensity of 100,000, and adduct ion; the peak area was quantitated at the same time. Following that, the molecular ion peak and fragment ions were used for molecular formula prediction and alignment with the mzCloud, mzVault, and MassList databases to identify metabolites. The metabolites with coefficient of variation <30% in QC samples were retained as the final identification result and subsequent analysis was performed. The CD3.1 software was used for integration of detected chromatography peaks; the peak area of each characteristic peak represents a relative quantitation value for a metabolite. The total peak area was used to standardize the quantitation result to finally obtain the quantitation result of the metabolite. Following that, QC was performed on the data to ensure the accuracy and reliability of the data results. Multiple statistical analyses were performed on metabolites, including principal component analysis (PCA) and partial least squares discriminant analysis (PLS-DA) to reveal metabolism mode differences between different groups. Metabolite correlation analysis was used to reveal the relationships between samples and between metabolites. Finally, metabolic pathway function analysis was used to explain the biological significance of metabolite correlation. In this experiment, *p* ≤ 0.05 and (variable importance of projection) VIP > 1 were used to screen for potential biomarkers. Differentially expressed metabolites with fold change (FC) >1.25 or <0.80 were used for metabolic pathway enrichment analysis based on KEGG data (https://www.kegg.jp/).

### 2.19. Statistical Analysis

SPSS 22.0 statistical software was used for statistical analysis, and data was expressed as mean ± standard deviation. One-way ANOVA was used for intergroup comparison. A difference of *p* < 0.05 was considered to be statistically significant.

## 3. Results

### 3.1. Therapeutic Effects of XCH on Mice with Viral Pneumonia

**Figure 1:**
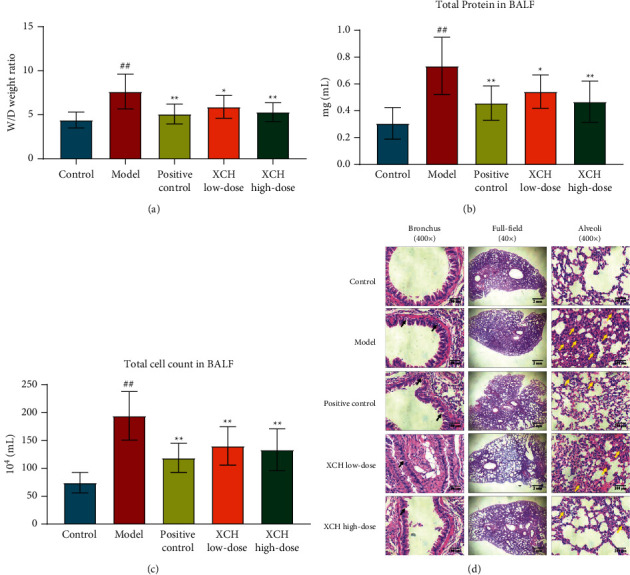
XCH ameliorated viral pneumonia induced by poly (I:C). (a) XCH treatment decreased the *W*/*D* ratio in lung tissue. (b) XCH treatment decreased the total protein concentration in BALF. (c) XCH treatment decreased the total cell count in BALF. (d) H&E staining indicated that XCH treatment alleviated the pathological changes in lung (40 and 400×). Black arrows indicated the damage in bronchial epithelial structure and yellow arrows indicated the infiltration of inflammatory cells. Control, model, positive control, XCH low-dose, and XCH high-dose groups (*n* = 10 per group). ^##^: *p* < 0.01 compared with the control group; ^*∗*^: *p* < 0.05 compared with the model group; ^*∗*^^*∗*^: *p* < 0.01 compared with the model group.

The *W*/*D* ratio of mouse lung tissues in the model group was significantly elevated (*p* < 0.01) compared with that in the control group. However, treatment with either DXM or the high dose of XCH significantly decreased the *W*/*D* ratio (both *p* < 0.01) compared with that in the model group (Figure 1(a)). The BALF total protein content was significantly increased in the model group (*p* < 0.01) compared with that of the control group and was significantly decreased in positive control and XCH low- and high-dose groups (*p* < 0.01, *p* < 0.05, and *p* < 0.01, respectively, Figure 1(b)). Compared with that in the control group, BALF total cell count was significantly increased in the model group (*p* < 0.01), and, compared with the model group value, treatment with low- and high-dose XCH and with DXM all significantly decreased BALF total cell count (all *p* < 0.01, Figure 1(c)). Lung H&E staining results showed intact bronchial epithelial structure, normal interalveolar septum, absence of interstitial edema in the lungs, and absence of significant inflammatory cell exudates in the control group. In the model group, bronchial epithelial structure was no longer intact and infiltration by a considerable number of inflammatory cells could be observed (Figure 1(d)). Treatment with DXM and low- and high-dose XCH significantly improved histopathological changes in lung tissues from mice with viral pneumonia.

### 3.2. Effects of XCH on Inflammatory Response in Mice with Viral Pneumonia

**Figure 2:**
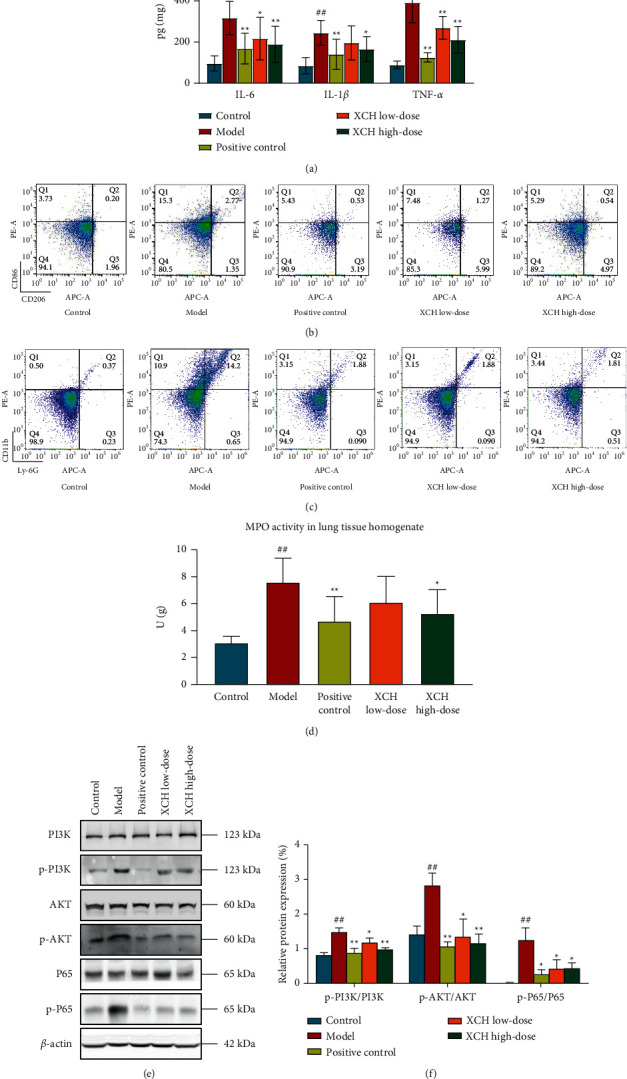
XCH reduced the inflammatory response in viral pneumonia model mice induced by poly (I:C). (a) XCH treatment decreased the levels of proinflammatory cytokines in BALF. (b) Flow cytometry showed that XCH treatment decreased the ratio between CD86^+^ and CD206^+^ cells in BALF. (c) Flow cytometry showed that XCH treatment decreased the proportion of Ly6G^+^CD11b^+^ cells in BALF. (d) XCH treatment decreased the activity of MPO in lung tissue homogenate. ((e) and (f)) Western blot showed that XCH treatment inhibited the activation of PI3K/AKT/NF-*κ*B signaling pathway. Control, model, positive control, XCH low-dose, and XCH high-dose groups (*n* = 10 per group). ^##^: *p* < 0.01 compared with the control group; ^*∗*^: *p* < 0.05 compared with the model group; ^*∗*^^*∗*^: *p* < 0.01 compared with the model group.

We used commercial ELISA kits to measure levels of inflammatory factors (IL-6, IL-1*β*, and TNF-*α*) in mouse BALF. Compared with those in the control group, the levels of IL-6, IL-1*β*, and TNF-*α* in BALF were significantly increased in the model group (all *p* < 0.01), and, compared with those in the model group, the levels of BALF IL-6 and TNF-*α* were significantly decreased following DXM and low- and high-dose XCH treatment (*p* < 0.01, *p* < 0.05, and *p* < 0.01 for IL-6, respectively, and *p* < 0.01 for all for TNF-*α*). Administration of DXM and high-dose XCH significantly decreased levels of IL-1*β* compared with the model group (*p* < 0.01 and *p* < 0.05, respectively, Figure 2(a)).

In addition, we also used flow cytometry to measure the expression of CD86 and CD206 in macrophages and of Ly6G and CD11b in BALF neutrophils from different groups. Compared with that in the control group, the ratio of CD86-expressing to CD206-expressing macrophages was increased (*p* < 0.01). The ratios of CD86-expressing to CD206-expressing macrophages were lower in positive control, XCH low-dose, and XCH high-dose groups compared with the model group (*p* < 0.01, *p* < 0.05, and *p* < 0.01, respectively, Figure 2(b) and Table 1). In addition, the proportion of Ly6G^+^ CD11b^+^ neutrophils in BALF was increased in model group as compared to the control group; DXM and low-dose and high-dose XCH treatment decreased the proportion of Ly6G^+^ CD11b^+^ neutrophils in BALF compared with the model group (all *p* < 0.01, Figure 2(c) and Table 2). The activity of MPO in lung tissue homogenate was also tested to observe the effects of XCH on neutrophil infiltration. Compared with the control group, the activity of MPO in model group was increased (*p* < 0.01). DXM and high dose of XCH treatment decreased the MPO activity as compared with the model group (*p* < 0.01 and *p* < 0.05, respectively, Figure 2(d)).

The activation of PI3K/AKT/NF-*κ*B signaling pathway in lung tissue in each group was measured using western blot. The phosphorylations of PI3K, AKT, and NF-*κ*B p65 were higher in model group as compared with those in the control group (all *p* < 0.01). DXM and low dose and high dose of XCH reduced the phosphorylations of PI3K (*p* < 0.01, *p* < 0.05, and *p* < 0.01, respectively), AKT (*p* < 0.01, *p* < 0.05, and *p* < 0.01, respectively), and NF-*κ*B p65 (all *p* < 0.05) as compared with the model group (Figures 2(e) and 2(f)).

Thus, a high dose of XCH could significantly improve pathological changes in lung tissues and inhibit inflammatory responses in viral pneumonia. Therefore, the high dose of XCH was selected for subsequent studies on the effects of XCH on serum metabolites in mice with viral pneumonia.

### 3.3. Effects of XCH on Serum Metabolite Levels in Mice with Viral Pneumonia

**Figure 3:**
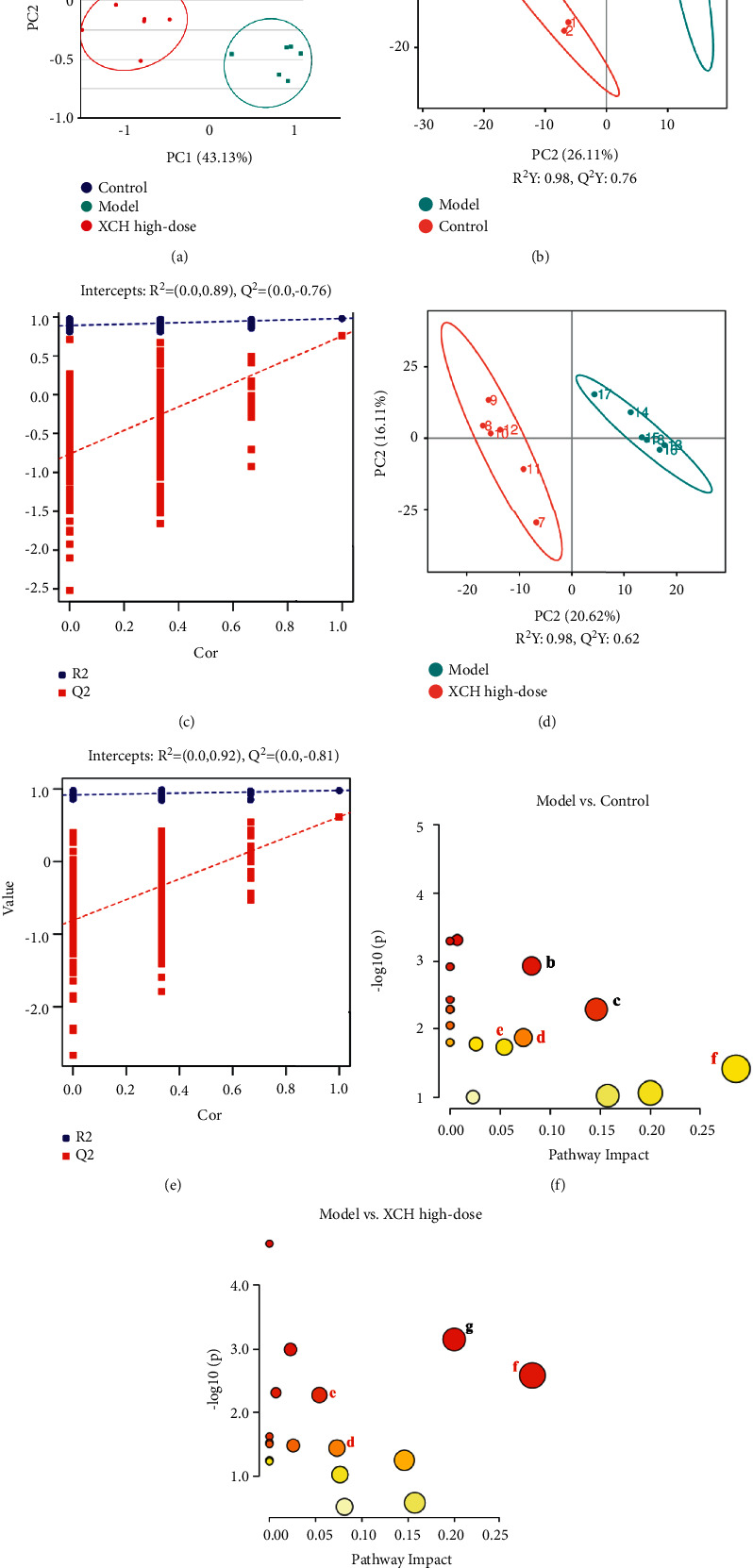
XCH treatment modulated the serum metabolites in viral pneumonia model mice induced by poly (I:C). (a) Scores plots of PCA among each group. ((b) and (c)) Scores plots of PLS-DA between the control and model groups and the corresponding coefficient of loading plots. ((d) and (e)) Scores plots of PLS-DA between the model and XCH high-dose groups and the corresponding coefficient of loading plots. ((f) and (g)) Summary of pathway analysis of serum samples between control and model groups (f) and between model and XCH high-dose groups (g); the same pathways were written in red. a: tyrosine metabolism; b: amino sugar and nucleotide sugar metabolism; c: starch and sucrose metabolism; d: arginine and proline metabolism; e: steroid hormone biosynthesis; f: taurine and hypotaurine metabolism; g: biotin metabolism. Control, model, and XCH high-dose groups (*n* = 6 per group).

Previous studies have shown that inflammation and injury are accompanied by changes in metabolic levels [18]. Untargeted metabolomics were used to elucidate drug intervention mechanisms. Many TCM herbs regulate endogenous metabolites to enable their therapeutic effects on viral pneumonia. An oral solution of Pu-Di-Lan has been shown to regulate levels of aspartate and L-cysteine and inhibit NF-*κ*B pathway activation to alleviate acute lung injury [19]. It was shown that *Lonicera japonica* and *Forsythia suspensa* could inhibit inflammatory responses in a mouse model of H1N1-induced pneumonia and simultaneously regulate the levels of galactose metabolism, glycine, serine, and threonine metabolism and synthesis and degradation of ketone bodies [20]. We further used untargeted metabolomics to study the effects of XCH on serum metabolites in mice with viral pneumonia. The PCA plot showed that the normal and model groups were well differentiated and that the model and XCH high-dose group were also well differentiated (Figure 3(a)). To identify differentially expressed metabolites, PLS-DA was used to analyze the model group and the explanatory power (*R*^2^) and predictive power (*Q*^2^) of the model group were assessed in the constructed PLS-DA model group. Compared with the control group, the model group had *R*^2^ = 0.89 and *Q*^2^ = −0.76, whereas XCH high-dose group had *R*^2^ = 0.92 and *Q*^2^ = −0.81 compared with the model group (Figures 3(b)–3(e)). These results showed that the model was stable and had good predictive power.

The two following criteria were used to screen for differentially expressed metabolites: *p* < 0.05 and VIP >1.0, where 25 differentially expressed metabolites were identified in total (Table 3). Compared with those in the control group, the serum levels of D-glucose 6-phosphate, homogentisic acid, D-ala-D-ala, hydroxyproline, corticosterone, prostaglandin A2, and arachidic acid were significantly increased, while those of ascorbic acid, choline, creatine, D-glucosamine, L-pipecolate, hypotaurine, gentisic acid, hydrocortisone, cortisone, L-adrenaline, kynurenic acid, pyroglutamic acid, taurocholic acid, and palmitoleic acid were significantly decreased in the model group. Serum levels of ascorbic acid, choline, biotin, creatine, L-pipecolate, hypotaurine, hydrocortisone, cortisone, taurocholic acid, and prostaglandin A2 were significantly increased in the XCH high-dose group compared with those in the model group, while those of cholic acid, creatinine, D-ala-D-ala, hydroxyproline, kynurenic acid, pyroglutamic acid, corticosterone, and arachidic acid were significantly decreased in the XCH high-dose group (Table 3).

### 3.4. Pathway Analysis of Differential Metabolites

The MetaboAnalyst platform was used for metabolic pathway enrichment analysis of differentially expressed metabolites in the model group together with the KEGG database. Differential metabolic pathways were selected based on a pathway impact >0.05 and *p* < 0.05. Differential metabolic pathways between the control group and model group included metabolism of tyrosine, amino sugar and nucleotide sugar, starch and sucrose, arginine and proline, and taurine and hypotaurine and biosynthesis of steroid hormone (Figure 3(f)). Differential metabolic pathways between the model group and the XCH high-dose group included metabolism of arginine and proline, taurine and hypotaurine, and biotin, as well as biosynthesis of steroid hormone (Figure 3(g)). Among these pathways, arginine and proline metabolism, steroid hormone biosynthesis, and taurine and hypotaurine metabolism were pathways common to the control, model, and XCH high-dose groups.

## 4. Discussion

Viral pneumonia is a lung interstitial and parenchymal inflammation caused when viruses invade the respiratory tract epithelium and alveolar epithelium [21]. Inflammation which is the main mechanism causing alveolar-capillary membrane damage and destruction of the alveolar-capillary barrier resulting in lung edema are important characteristics of viral pneumonia [22]. In this study, intratracheal injection of poly (I:C) was used to construct a mouse model of viral pneumonia. Measurement of lung tissue *W*/*D*, BALF protein concentration, and total cell count has been used to determine the severity of lung edema and alveolar-capillary permeability in mice with viral pneumonia [7, 23]. Our results showed that lung tissue *W*/*D*, BALF protein concentration, and total cell count were significantly increased in the mouse model of viral pneumonia, suggesting that alveolar-capillary permeability increased, and that lung edema was present in mice with viral pneumonia. Histopathology section observations were used to determine lung tissue injury in mice with viral pneumonia. The H&E staining showed large areas of inflammatory cell infiltration, bronchial epithelial structure disruption, lung parenchymal injury, severe alveolar destruction, alveolar cavity fusion, dilation and collapse of interalveolar septum, and interstitial edema in mice with viral pneumonia [15, 23]. The above results were consistent with the pathological presentation of viral pneumonia, suggesting that the modeling method of this study successfully simulated the main symptoms and pathological characteristics of viral pneumonia. The XCH intervention decreased lung tissue *W*/*D*, BALF protein concentration, and total cell count and improved pathological changes in lung tissues, suggesting that XCH has therapeutic effects in mice with viral pneumonia. DXM is a widely used anti-inflammatory agent in clinic and is commonly used as the positive drug in the study of viral pneumonia model induced by poly (I:C). In their study, Cui et al. measured the effects of andrographolide on viral pneumonia model mice induced by poly (I:C) and DXM was used as the positive control in this study [16]. Therefore, we chose DXM as the positive control in our study. Our result showed minimal differences in reducing alveolar-capillary permeability and improving pathological changes between XCH high-dose and positive control groups, suggesting that XCH may serve as alternative treatment to DXM on viral pneumonia.

In addition, we studied the effects of XCH on inflammatory response in mice with viral pneumonia. ELISA results showed that XCH treatment could decrease levels of BALF IL-6, IL-1*β*, and TNF-*α* in mice with viral pneumonia. Furthermore, XCH treatment could decrease the BALF CD86+/CD206+ cell ratio and simultaneously decrease the percentage of Ly6G + CD11b + cells. CD86 and CD206 are surface markers of M1 and M2 macrophages (M*φ*), respectively, and M*φ* can be classified into two major types based on the phenotype and secreted cytokines, that is, classically activated M*φ* (M1M*φ*) and alternatively activated M*φ* (M2M*φ*) [24]. M1M*φ* have a proinflammatory phenotype and can produce proinflammatory cytokines and have antimicrobial and tumor-killing activity. These macrophages are mainly activated by lipopolysaccharide (LPS) or interferon-*γ* [25, 26]. During the acute phase, activated M1M*φ* secrete large amounts of proinflammatory mediators such as TNF-*α* and IL-1*β* [27]. M2M*φ* are induced by IL-4 or IL-13 and produce various anti-inflammatory cytokines, such as IL-10 and argininase-1 (ARG-1) to alleviate inflammation and promote wound healing [28, 29]. Besides M*φ*, neutrophils are also vital to the occurrence and progression of inflammation. Alveolar macrophages (AM*φ*) and neutrophils are both phagocytes in the innate immune system. Neutrophils express Ly6G and CD11b, phagocytose pathogens, produce reactive oxygen species and toxic enzymes (such as elastase), or produce neutrophil extracellular traps (NETs) and therefore play an important role in innate immunity [30]. Infection by a pathogen can cause overactivation of neutrophils and secretion of a large number of NETs. Excessive NETs can induce M1 macrophage polarization, further causing cytokine storm and progression of acute lung injury [31]. We have also tested the activity of myeloperoxidase (MPO) in lung tissue to evaluate the neutrophil infiltration after XCH treatment. XCH treatment also decreased the MPO activity in lung tissue homogenate. MPO is highly expressed in neutrophil and is an important marker of activated neutrophil. The MPO activity was increased in pneumonia model mice. Investigating the activity of MPO could reflect the infiltration of neutrophils [32].

In addition, our results showed that XCH treatment reduced the phosphorylation of PI3K, AKT, and NF-*κ*B p65 in lung, indicating that XCH could inhibit the activation PI3K/AKT/NF-*κ*B signaling pathway in viral pneumonia model mice. PI3K/AKT/NF-*κ*B signaling pathway plays an important role during the activation of inflammatory response. Viral infection can induce the phosphorylation and activation of PI3K [33]. Then, activated PI3K can cause the conversion of phosphatidylinositol 4,5-bisphosphate (PIP2) to phosphatidylinositol-3,4,5-trisphosphate (PIP3). PIP3 can interact with the PH domain of AKT and trigger the phosphorylation of AKT. Phosphorylated AKT can induce the phosphorylation and activation of subunit p65 of NF-*κ*B. NF-*κ*B is an important nuclear transcription factor, activated NF-*κ*B p65 can enter the nucleus and induce the transcription of several proinflammatory genes such as IL-6, IL-1*β*, and TNF-*α* and contribute to the progression of inflammatory response [34].

We also used untargeted metabolomics to study the effects of XCH on serum metabolites in mice with viral pneumonia. PCA and PLS-DA results showed significant changes in serum metabolism in mice with viral pneumonia and also showed that XCH intervention could significantly affect these changes. Furthermore, differentially expressed metabolite analysis showed that XCH could affect the levels of 25 metabolites such as ascorbic acid, D-glucose 6-phosphate, choline, and creatine. Treatment with XCH decreased the levels of cholic acid, creatinine, D-ala-D-ala, and hydroxyproline and increased those of ascorbic acid, choline, biotin, and creatine. Metabolic pathway analysis of differentially expressed metabolites via MetaboAnalyst demonstrated changes in arginine and proline metabolism, steroid hormone biosynthesis, and taurine and hypotaurine metabolism occurring in the control, model, and XCH groups, suggesting that XCH treatment may regulate these pathways to carry out the associated therapeutic effects in viral pneumonia.

### 4.1. Arginine and Proline Metabolism

Our study results showed that XCH intervention could increase the level of creatine and decrease that of hydroxyproline, which are compounds associated with arginine and proline metabolism in mice with viral pneumonia. Arginine is a semiessential amino acid for humans which participates in many metabolic processes, is present in many proteins, and plays an important role in immune responses [35]. Evidence has shown that arginine supplementation can lead to T cell proliferation and eliminate T cell suppression after trauma [36, 37]. Creatine is a downstream product of arginine and an important energy source. A recent study found that creatine promotes ARG-1 expression to inhibit induction of immune effectors such as iNOS, thereby maintaining the balance between M1 and M2 macrophages and ultimately regulating macrophage-mediated immune responses [38]. M1 macrophages mainly metabolize arginine through NOS2 while simultaneously inhibiting ARG-1 expression [39]. M2 macrophages mainly metabolize arginine through ARG-1, although several M2 macrophage functions are mediated by proline [40, 41]. Proline and its metabolite hydroxyproline are derivatives of arginine and are key regulators in many physiological and biochemical processes in cells [42]. ARG has been proven to alleviate inflammation in addition to its immunomodulatory characteristics [43]. ARG injection in sepsis patients was shown to improve the health status of patients [44], and arginine pretreatment alleviated liver injury in a rat model of ischemia-reperfusion [45]. However, arginine has complex negative effects in blood vessels. ARG degrades arginine to ornithine and urea [46], which are precursors for collagen and proline synthesis and have important functions in vascular remodeling [47, 48]. Arginine can also be metabolized by NOS to form citrulline and nitric oxide, a vasodilator. Therefore, the effector mechanism of XCH in regulating macrophage polarization in mice with viral pneumonia may be related to arginine and proline metabolism. In future studies, *in vitro* experiments can be used to elucidate the effector mechanism of XCH on macrophage polarization from the perspective of arginine and proline metabolism.

### 4.2. Steroid Hormone Biosynthesis

Our results showed that levels of serum cortisone and hydrocortisone were decreased and those of corticosterone were increased in mice with viral pneumonia. After XCH intervention, the levels of cortisone and hydrocortisone increased, while those of corticosterone decreased. Steroids are endogenous substances involved in endocrine system of mammals and have distinct roles in life maintenance and immune regulation [49]. Steroid hormones include adrenocortical hormones and androgens and estrogen and progesterone [50]. Adrenocortical hormones are mainly secreted by adrenal glands and include glucocorticoids and mineralocorticoids [51]. Steroid biosynthesis starts when cholesterol moves from cellular reserves to the mitochondrial inner membrane and cytochrome P450 cholesterol side chain cleavage (P450cc or CYP11A1) occurs [52–55]. As steroids have significantly anti-inflammatory and immunosuppressant effects, they are widely used to treat inflammation, autoimmune disease, and cancer and are the best-selling drugs after antibiotics [56]. Viral pneumonia can cause hyperinflammation, and the anti-inflammatory properties of steroids make these an effective treatment option. Cortisone is a glucocorticoid secreted by adrenal glands based on the circadian rhythm and is itself inactive. Cortisone needs to be metabolized in the body to hydrocortisone (also known as cortisol) to enable its effects [57]. As natural glucocorticoids have a short half-life, some semisynthetic glucocorticoids such as prednisone, prednisolone, methylprednisolone, triamcinolone, and dexamethasone have been obtained by structural modification of the cortisone backbone, which increases their half-lives and activity [58–60]. A clinical study has shown that steroids could stabilize hemodynamics and shorten the length of intensive care unit stay and duration of mechanical ventilation in patients with septic shock [61]. The treatment guidelines for COVID-19 mentioned that low-dose steroids can alleviate SARS-CoV-2-induced cytokine storm and decrease peripheral vasodilation [62]. In the substudy of the Corti-TC trial [63], blood cortisol levels could be used as a marker in hospital-acquired pneumonia to decide whether cortisol treatment is required [64]. Although steroid treatment has potent effects in inflammation, some data indicates that long-term steroid treatment in viral pneumonia may cause side effects, such as an increase in the mortality rate [65].

### 4.3. Taurine and Hypotaurine Metabolism

Our metabolomic analysis results showed that levels of hypotaurine and taurine in the serum decreased in mice with viral pneumonia and that XCH intervention could increase these metabolites. Taurine is a sulfur-containing amino acid that is ubiquitous in mammals and has important biological functions such as regulating osmotic pressures, stabilizing cell membranes, and regulating intracellular calcium levels [66]. Recent studies found that taurine has pharmacological effects such as delaying obesity, decreasing blood lipid levels, and improving diabetes as well as having antitumor effects [67–69]. In addition, the immune-boosting and anti-inflammatory functions of taurine are common mechanisms that benefit the control of these chronic diseases. *In vivo* taurine levels will decrease when certain diseases occur. For example, plasma taurine levels were 41% lower in obese volunteers compared with those in healthy volunteers [70]. In a clinical study on 398 women with singleton pregnancies, an increase in plasma taurine level was significantly correlated with a decreased risk of gestational diabetes [71]. In animal models of LPS-induced acute lung injury, taurine intervention could decrease inflammation and oxidative stress in lung [72]. Combination therapy with taurine and dexmedetomidine inhibited p65 phosphorylation and p65 nuclear translocation to significantly inhibit NF-*κ*B pathway activation [73]. Hypotaurine is a precursor of taurine and undergoes oxidation to form taurine [74]. Metabolomic analysis of acute liver failure patients found that levels of hypotaurine and taurine were increased among liver metabolites, while the total level of reduced and oxidized glutathione was decreased [75].

In conclusion, our study demonstrated that treatment with XCH can ameliorate viral pneumonia and reduce inflammatory response in viral pneumonia. The mechanism of action of XCH in the treatment of viral pneumonia may be associated with inhibiting the activation of PI3K/AKT/NF-*κ*B signaling pathway in lung and regulating arginine and proline metabolism, steroid hormone biosynthesis, and taurine and hypotaurine metabolism in serum.

## Figures and Tables

**Table 1:** Effects of XCH on the proportions of CD86^+^ and CD206^+^ cells in BALF.

Group	CD86^+^ (%)	CD206^+^ (%)	CD86^+^/CD206^+^
Control	2.95 ± 0.09	1.54 ± 0.18	1.94 ± 0.17
Model	12.91 ± 0.91^##^	2.24 ± 0.27^##^	5.82 ± 0.74^##^
Positive control	4.57 ± 0.62^*∗*^^*∗*^	2.65 ± 0.49	1.83 ± 0.46^*∗*^^*∗*^
XCH low-dose	9.83 ± 0.48^*∗*^^*∗*^	2.04 ± 0.2	4.88 ± 0.92^*∗*^
XCH high-dose	5.49 ± 2.03^*∗*^^*∗*^	2.53 ± 0.44	2.28 ± 1.05^*∗*^^*∗*^

Control, model, positive control, XCH low-dose, and XCH high-dose groups (*n* = 10 per group). ^##^: *p* < 0.01 compared with the control group; ^*∗*^: *p* < 0.05 compared with the model group; ^*∗*^^*∗*^: *p* < 0.01 compared with the model group.

**Table 2:** Effects of XCH on the proportion of Ly6G^+^CD11b^+^ cells in BALF.

Group	Ly6G^+^CD11b^+^ (%)
Control	0.383 ± 0.03
Model	6.368 ± 0.59^##^
Positive control	2.44 ± 0.12^*∗*^^*∗*^
XCH low-dose	0.997 ± 0.19^*∗*^^*∗*^
XCH high-dose	0.681 ± 0.1^*∗*^^*∗*^

Control, model, positive control, XCH low-dose, and XCH high-dose groups (*n* = 10 per group). ^##^: *p* < 0.01 compared with the control group; ^*∗*^^*∗*^: *p* < 0.01 compared with the model group.

**Table 3:** Differential metabolites in viral pneumonia model mice induced by poly (I:C) after the treatment of XCH.

No.	Formula	RT (min)	*m/z*	Metabolites	VIP		FC		Trend		Pathway
					M vs. C	X vs. M	M vs. C	X vs. M	M vs. C	X vs. M	
1	C_6_H_8_O_6_	1.65	175.02	Ascorbic acid	1.44	1.71	0.64	1.30	↓^##^	↑^*∗*^	
2	C_6_H_13_O_9_P	1.42	259.02	D-glucose 6-phosphate	1.94	1.44	1.95	0.68	↑^##^	↓	c
3	C_5_H_13_NO	1.33	104.11	Choline	1.46	1.84	0.65	1.50	↓^#^	↑^*∗*^	
4	C_10_H_16_N_2_O_3_S	5.35	245.10	Biotin	1.91	2.27	0.47	1.79	↓	↑^*∗*^^*∗*^	g
5	C_6_H_13_O_9_P	1.53	259.02	D-mannose 6-phosphate	1.87	1.62	0.73	1.36	↓	↑	b
6	C_4_H_9_N_3_O_2_	1.41	132.08	Creatine	1.53	1.44	0.68	1.32	↓^#^	↑^*∗*^	d
7	C_6_H_13_NO_5_	1.43	180.09	D-glucosamine	2.12	1.24	0.47	1.17	↓^##^	↑	b
8	C_6_H_11_NO_2_	1.15	130.09	L-pipecolate	2.09	2.10	0.60	1.53	↓^##^	↑^*∗*^	
9	C_2_H_7_NO_2_S	1.35	110.03	Hypotaurine	1.71	1.55	0.68	1.32	↓^#^	↑^*∗*^	f
10	C_8_H_8_O_4_	5.08	167.03	Homogentisic acid	1.55	1.07	1.95	1.33	↑^##^	↑	a
11	C_7_H_6_O_4_	5.64	153.02	Gentisic acid	2.34	1.30	0.41	1.73	↓^##^	↑	a
12	C_24_H_40_O_5_	6.61	407.28	Cholic acid	1.15	1.91	1.14	0.17	↑	↓^*∗*^^*∗*^	
13	C_21_H_30_O_5_	5.59	363.22	Hydrocortisone	1.64	1.69	0.54	1.91	↓^#^	↑^*∗*^^*∗*^	e
14	C_21_H_28_O_5_	5.85	361.20	Cortisone	1.76	1.24	0.58	1.46	↓^#^	↑^*∗*^	e
15	C_9_H_13_NO_3_	5.56	184.10	L-adrenaline	1.20	2.37	0.62	1.27	↓^#^	↑	a
16	C_4_H_7_N_3_O	1.38	114.07	Creatinine	1.72	1.30	1.16	0.66	↑	↓^*∗*^	
17	C_6_H_12_N_2_O_3_	1.44	161.09	D-Ala-D-Ala	2.04	1.34	1.68	0.38	↑^##^	↓^*∗*^^*∗*^	
18	C_5_H_9_NO_3_	1.35	132.07	Hydroxyproline	1.49	1.41	1.43	0.42	↑^#^	↓^*∗*^^*∗*^	d
19	C_10_H_7_NO_3_	5.38	190.05	Kynurenic acid	2.00	1.18	0.45	0.60	↓^##^	↓^*∗*^	
20	C_5_H_7_NO_3_	6.39	130.05	Pyroglutamic acid	1.99	1.43	0.34	0.57	↓^##^	↓^*∗*^^*∗*^	
21	C_21_H_30_O_4_	6.25	347.22	Corticosterone	1.48	1.45	1.57	0.53	↑^##^	↓^*∗*^^*∗*^	e
22	C_26_H_45_NO_7_S	7.72	516.30	Taurocholic acid	1.69	2.05	0.25	1.87	↓^#^	↑^*∗*^^*∗*^	f
23	C_20_H_30_O_4_	7.37	333.21	Prostaglandin A2	1.82	1.21	1.32	1.56	↑^#^	↑^*∗*^^*∗*^	
24	C_20_H_40_O_2_	11.56	311.30	Arachidic acid	1.69	1.69	1.40	0.26	↑^##^	↓^*∗*^^*∗*^	
25	C_16_H_30_O_2_	7.85	255.23	Palmitoleic acid	1.48	1.62	0.62	0.87	↓^##^	↓	

Control, model, and XCH high-dose groups (*n* = 6 per group). RT: retention time; VIP: variable importance of projection; FC: fold change;^#^: *p* < 0.05 as compared to the control group; ^##^: *p* < 0.01 as compared to the control group; ^*∗*^: *p* < 0.05 as compared to the model group; ^*∗*^^*∗*^: *p* < 0.01 as compared to the model group; ↑: content increased; ↓: content decreased; vs.: versus; C: control group; M: model group; X: XCH high-dose group; a: tyrosine metabolism; b: amino sugar and nucleotide sugar metabolism; c: starch and sucrose metabolism; d: arginine and proline metabolism; e: steroid hormone biosynthesis; f: taurine and hypotaurine metabolism; g: biotin metabolism.

## Data Availability

The datasets used and/or analyzed during this study are available from the corresponding author upon reasonable request.

## Supplementary Materials

Supplementary Materials Figure S1: the chemical profiles of XCH using HPLC-MS. (a, b) The total ion chromatogram (TIC) in positive (a) and negative ion modes (b). (c–e) The main bioactive compounds of baicalin (c), saikosaponin A (d), saikosaponin D (e). The bioactive compounds detected in XCH were in red peaks and were confirmed by the reference standards in black peaks. Table S1: the characteristic fragment ions of reference standards in XCH.
