# Supplementary material for: Xiao-Chai-Hu Decoction Ameliorates Poly (I:C)-Induced Viral Pneumonia through Inhibiting Inflammatory Response and Modulating Serum Metabolism
Source: Evid Based Complement Alternat Med. 2022 Jul 12;2022:1240242. doi: 10.1155/2022/1240242 (PMC9296287; doi:10.1155/2022/1240242)
Supplement: Supplementary Materials — Figure S1: the chemical profiles of XCH using HPLC-MS. (a, b) The total ion chromatogram (TIC) in positive (a) and negative ion modes (b). (c–e) The main bioactive compounds of baicalin (c), saikosaponin A (d), saikosaponin D (e). The bioactive compounds detected in XCH were in red peaks and were confirmed by the reference standards in black peaks. Table S1: the characteristic fragment ions of reference standards in XCH. [file 1240242.f1.docx]

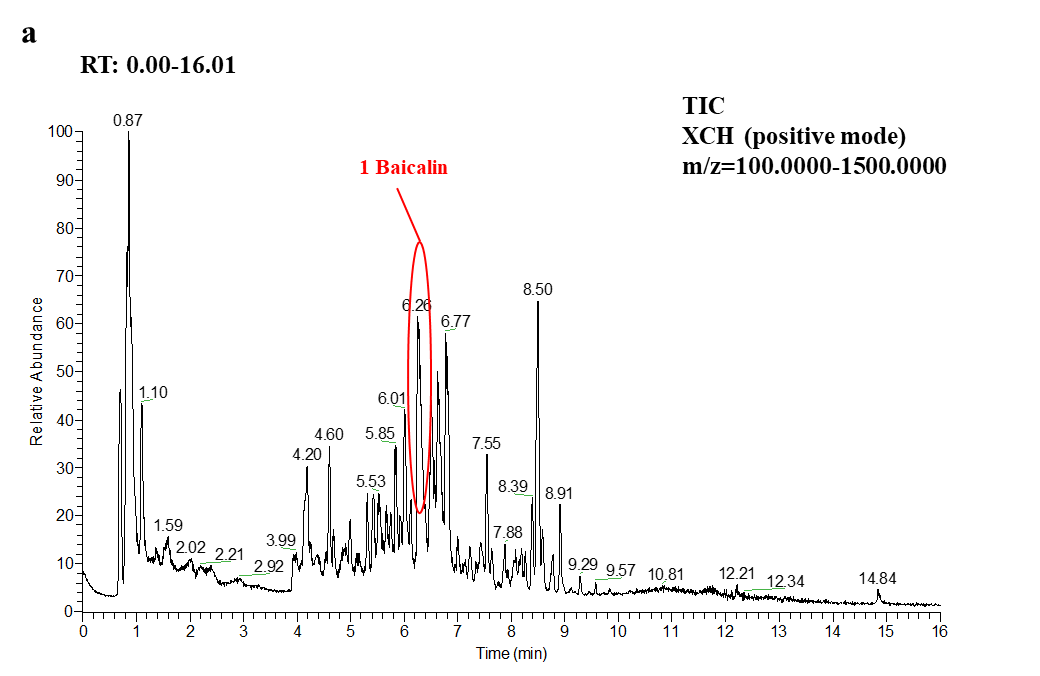


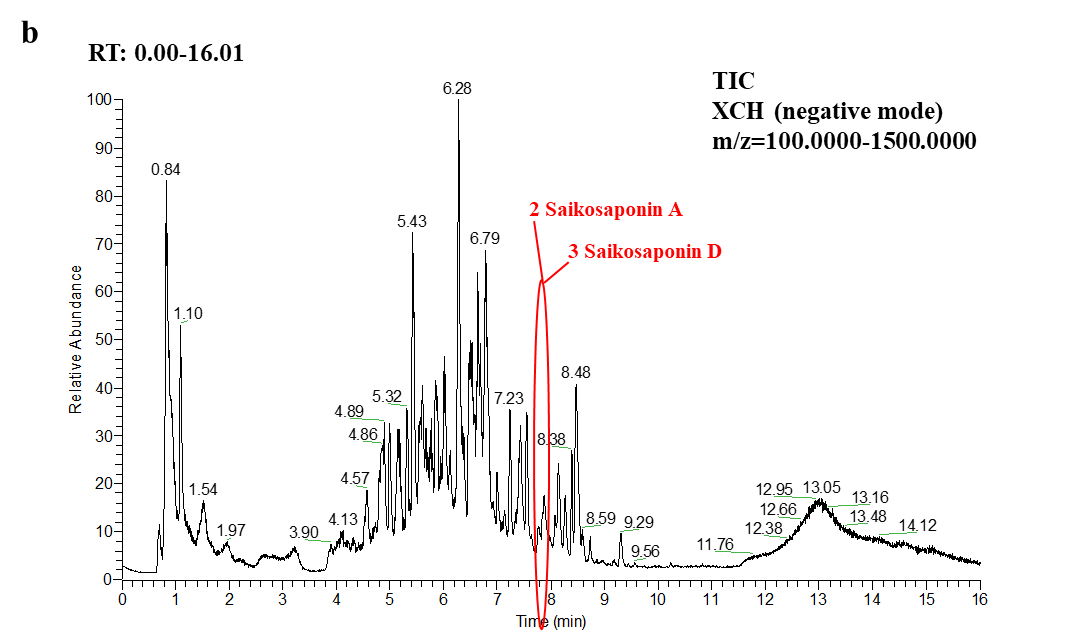


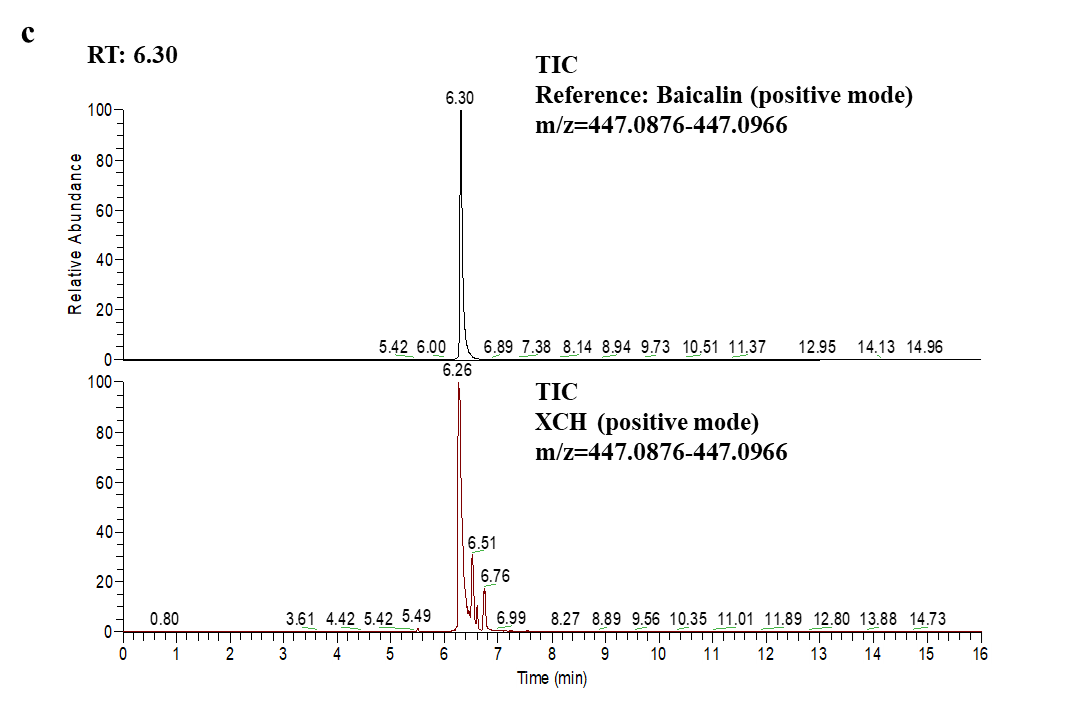


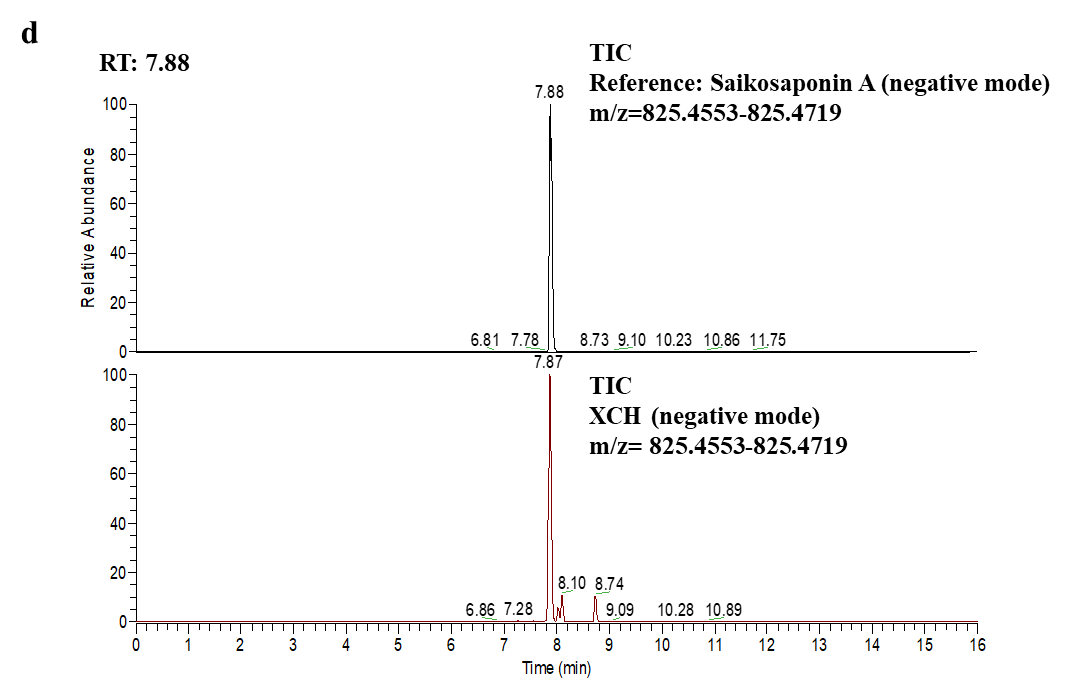


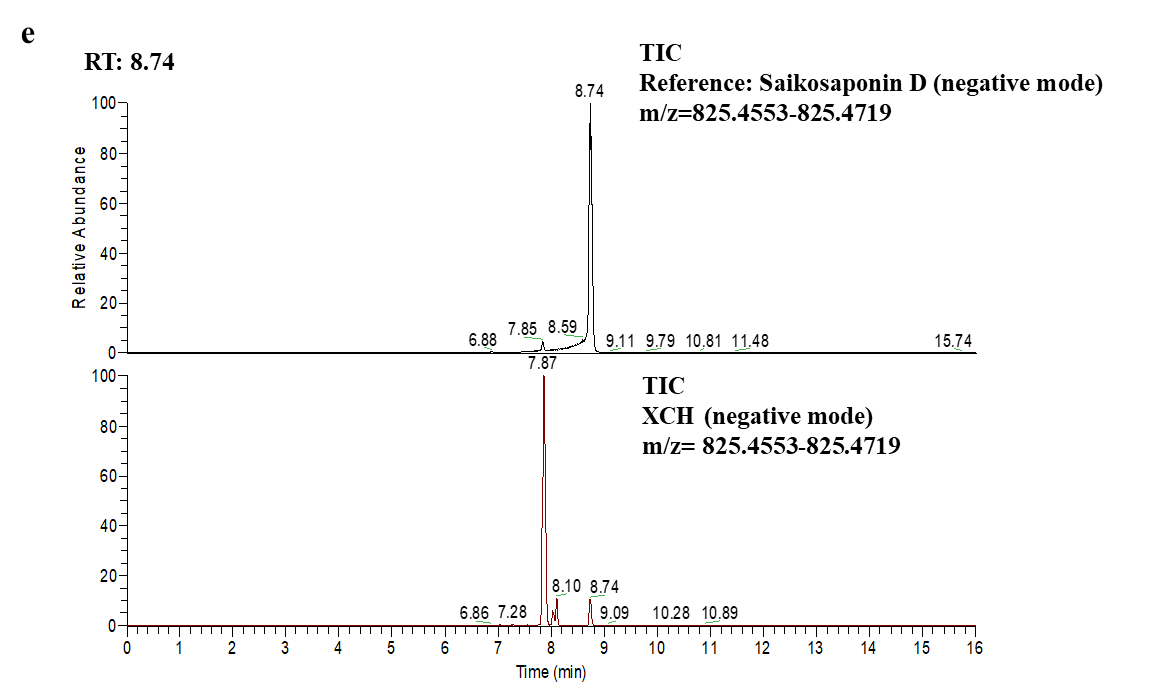


**Figure S1:** The chemical profiles of XCH using HPLC-MS. (**a, b**) The total ion chromatogram (TIC) in positive (**a**) and negative ion modes (**b**). (**c-e**) The main bioactive compounds of baicalin (**c**), , saikosaponin A (**d**) saikosaponin D (**e**),. The bioactive compounds detected in XCH were in red peaks and were confirmed by the reference standards in black peaks.

**TABLE S1 The characteristic fragment ions of reference standards in XCH**

| **Marking**  **peak no.** | **Name** | **RT**  **(min)** | **Ion** | **m/z** | **Herbs** |
| --- | --- | --- | --- | --- | --- |
| 1 | Baicalin | 6.30 | [M+H]^+^ | 447.0921 | *Scutellariae baicalensis* Georgi |
| 2 | Saikosaponin A | 7.86 | [M-H]^-^ | 779.4582 | *Bupleurum chinense* DC. |
| 3 | Saikosaponin D | 8.72 | [M-H]^-^ | 779.4582 | *Bupleurum chinense* DC. |
